# Chronic Irreducible Anterior Shoulder Dislocation With Axillary Vein Compression: A Case Report Highlighting Multidisciplinary Management With Reverse Total Shoulder Arthroplasty

**DOI:** 10.1155/cro/5089319

**Published:** 2025-10-08

**Authors:** Julio Nerys-Figueroa, Kai Zhu, Mahdi Mazeh, Chimdi Obinero, Wade Wines, Stephanie J. Muh

**Affiliations:** Department of Orthopaedic Surgery, Henry Ford Health, Detroit, Michigan, USA

**Keywords:** axillary vein compression, chronic dislocation, CTA, PROMIS, reverse shoulder arthroplasty, shoulder dislocation, thrombosis, vascular injury

## Abstract

**Introduction:**

Anterior shoulder dislocations are the most common major joint dislocation encountered in clinical practice. Although typically reducible, chronic irreducible anterior dislocations are rare and pose significant diagnostic and management challenges. Contributing factors include rotator cuff pathology, soft tissue interposition, and bony fragments. Vascular complications, while infrequent, must also be considered, particularly in chronic presentations.

**Case Report:**

We report the case of a 61-year-old female who sustained an anterior shoulder dislocation after falling on ice 8 weeks prior to presentation. She had a history of a previously canceled rotator cuff repair 3 years earlier. Multiple closed reduction attempts in the emergency department and operating room were unsuccessful. Magnetic resonance imaging (MRI) revealed a chronic massive rotator cuff tear with subscapularis interposition and the humeral head abutting the neurovascular bundle. Computed tomography angiography (CTA) demonstrated axillary vein compression without contrast opacification distal to the axilla. The patient was evaluated by vascular surgery prior to intervention, and an in-clinic venous duplex ultrasound confirmed the chronicity of the occlusive thrombus. Due to the risk of intraoperative complications, a vascular surgeon remained on standby during arthroplasty. Given the irreducibility, chronicity of rotator cuff pathology, and vascular involvement, a reverse total shoulder arthroplasty (rTSA) was performed with vascular surgery support. The patient experienced pain relief and modest improvements in range of motion and Patient-Reported Outcomes Measurement Information system (PROMIS) outcomes postoperatively, though persistent limitations necessitated a revision rTSA.

**Conclusion:**

This case underscores the importance of considering vascular compression in chronic irreducible shoulder dislocations. While axillary artery injury is well-documented, chronic axillary vein compression and thrombosis remain underreported. Advanced imaging, particularly CTA, should be utilized when the humeral head lies in close proximity to vascular structures. Such complex cases require management in a tertiary care setting with access to vascular surgery. Multidisciplinary planning, timely diagnosis, and appropriate surgical intervention are essential to optimize outcomes in these high-risk patients.

## 1. Introduction

Glenohumeral joint dislocations are common orthopedic injuries, with the anterior subtype being the most common [1]. Chronic irreducible anterior shoulder dislocations are rare, accounting for less than 2% of all shoulder dislocations [2]. Several factors can contribute to the irreducibility of the shoulder joint, such as cartilage deterioration, poor bone quality, and compromised soft tissues that can potentially compromise nearby structures and require open reduction [2].

Anterior shoulder dislocations may compromise adjacent structures such as the axillary artery, brachial plexus, and rotator cuff [3]. The incidence of axillary artery injury from shoulder glenohumeral dislocation is roughly 1%–2% [4]. However, to the author's knowledge, there have not been many published cases of shoulder dislocations that cause damage to the axillary vein.

The axillary vein is a major vein of the upper extremity (UE) that runs medially to the axillary artery [5]. Axillary vein compression can lead to complications such as deep vein thrombosis, UE swelling, and venous insufficiency [6, 7]. While axillary artery injuries are more commonly discussed in the context of shoulder dislocations, axillary vein compression is significantly underreported and may be clinically silent or misattributed.

Anteroposterior (AP) radiographic imaging is taken for diagnostic confirmation of dislocation and the assessment of associated osseous injuries [8]. Diagnosis of venous compression can be difficult as some patients might be asymptomatic, and when they do have symptoms such as pain and swelling, it can be nonspecific [9]. Imaging modalities used for diagnosis of venous compression includes ultrasound (US) with Doppler, contrast-enhanced computed tomography (CT), computed tomography venography (CTV), magnetic resonance imaging (MRI), and magnetic resonance venography (MRV) [9].

This article reports the case of an irreducible anterior shoulder dislocation due to an interposed subscapularis tendon with compression of the axillary vein that was treated with a reverse total shoulder arthroplasty (rTSA). To our knowledge, this is one of the few documented cases of chronic irreducible anterior shoulder dislocation complicated by axillary vein compression, requiring preoperative vascular imaging and multidisciplinary surgical planning.

## 2. Case Presentation

The patient, a 61-year-old female, sustained a fall on ice 8 weeks prior to presentation. Notably, she had a history of a previously cancelled rotator cuff repair 3 years earlier. She initially presented to the emergency department, where multiple closed reduction attempts were performed under sedation using sustained traction and counter-traction techniques, all of which were unsuccessful. She subsequently underwent an additional attempted closed reduction under general anesthesia in the operating room 2 weeks prior to her orthopedic clinic visit. On presentation to our clinic, physical examination revealed right shoulder pain, limited range of motion, and a visible deformity, likely accentuated by the patient's thin body habitus.

**Figure 1:**
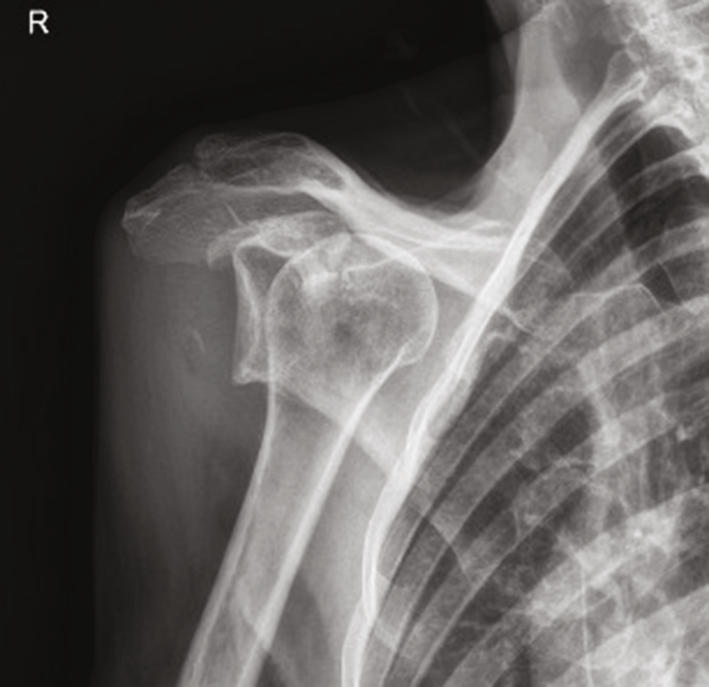
Pre-reduction Grashey radiograph of the right shoulder demonstrating anterior dislocation of the humeral head. No associated fracture is seen.

**Figure 2:**
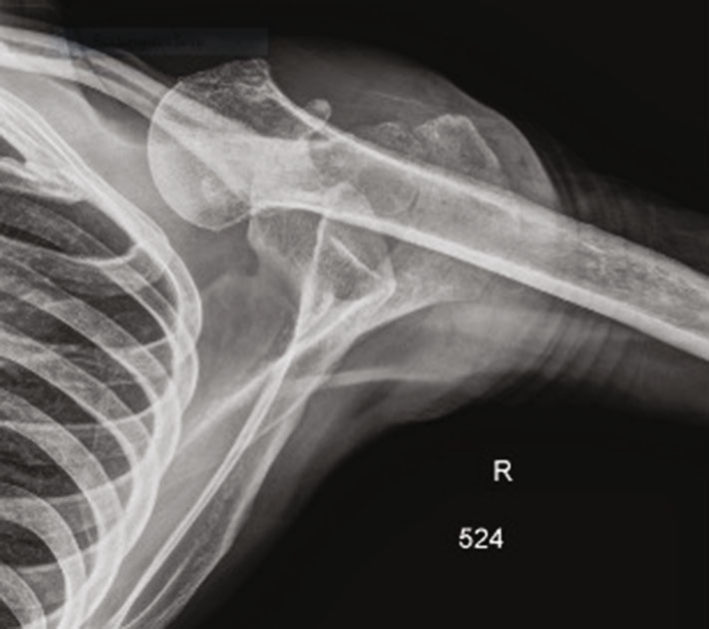
Pre-reduction axillary radiograph of the right shoulder showing anterior dislocation with medial migration of the humeral head relative to the glenoid. No associated fracture is seen.

**Figure 3:**
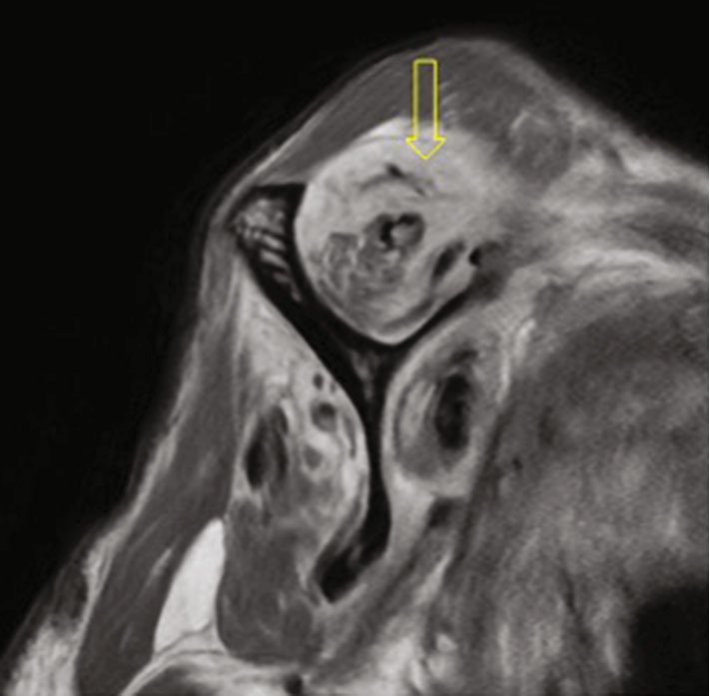
Sagittal T2-weighted MRI; the yellow arrow demonstrates atrophy and fatty infiltration of the right supraspinatus muscle, consistent with a chronic rotator cuff tear.

**Figure 4:**
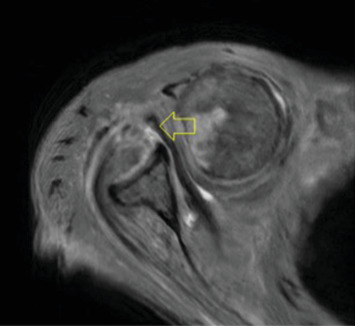
Axial T2-weighted MRI showing anterior dislocation of the right humeral head; the yellow arrow demonstrates interposition of the subscapularis tendon between the humeral head and glenoid.

**Figure 5:**
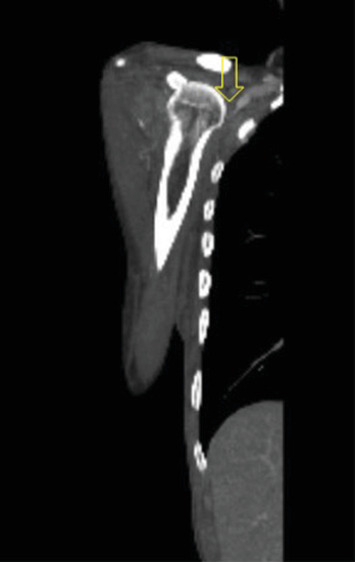
Contrast-enhanced CTA of the right upper extremity; the yellow arrow demonstrates compression of the axillary vein with absence of contrast opacification distal to the axilla.

Pre-reduction of the right shoulder in the Grashey and axillary views confirmed an anterior shoulder dislocation (Figures 1 and 2). An initial MRI examination of the shoulder was conducted, which revealed several significant findings. These included a chronic massive rotator cuff tear with evidence of fat atrophy (Figure 3). Importantly, the MRI confirmed the presence of an anterior shoulder dislocation with an interposed subscapularis tendon and a humeral head positioned near the neurovascular bundle (Figure 4). A contrast-enhanced computer tomography angiography (CTA) was performed and demonstrated compression of the axillary vein, with no contrast opacification of the veins below the axilla (Figure 5). The right subclavian, axillary, and UE arteries were patent.

Considering the irreducible nature of the anterior shoulder dislocation and the presence of axillary vein compression, a decision was made to proceed with surgical intervention. Given the chronicity of the rotator cuff tear, the degree of muscle atrophy, and the failure of closed reduction, rTSA was selected as the most appropriate treatment option. Traditional rotator cuff repair was not feasible due to severe tendon retraction [10]. This included an office visit with a vascular surgeon for preoperative planning, including a right UE venous duplex, which demonstrated totally occluding chronic deep vein thrombosis of the right axillary vein with partially occluding chronic deep vein thrombosis of the right mid and mid/distal brachial vein. The chronic thrombotic findings further emphasized the need for intraoperative vascular readiness, as unrecognized vessel compromise could increase the risk of perioperative complications. The patient was positioned in the beach chair position in the operating room, as documented in the operative note. General anesthesia with endotracheal intubation was administered uneventfully. A vascular surgeon was also on standby to intraoperatively to manage any unforeseen vascular complications. This precaution ensured the surgical team was prepared for any emergent vascular intervention, particularly if intraoperative contrast evaluation or decompression had become necessary. Intraoperatively, dissection was complicated by dense adhesions and significant anterior glenoid bone loss. These findings confirmed the chronic nature of the pathology and justified the need for prosthetic reconstruction. Additionally, conjoined tendon was noted to be displaced transversely, and the humeral head was noted to be subcoracoid.

**Figure 6:**
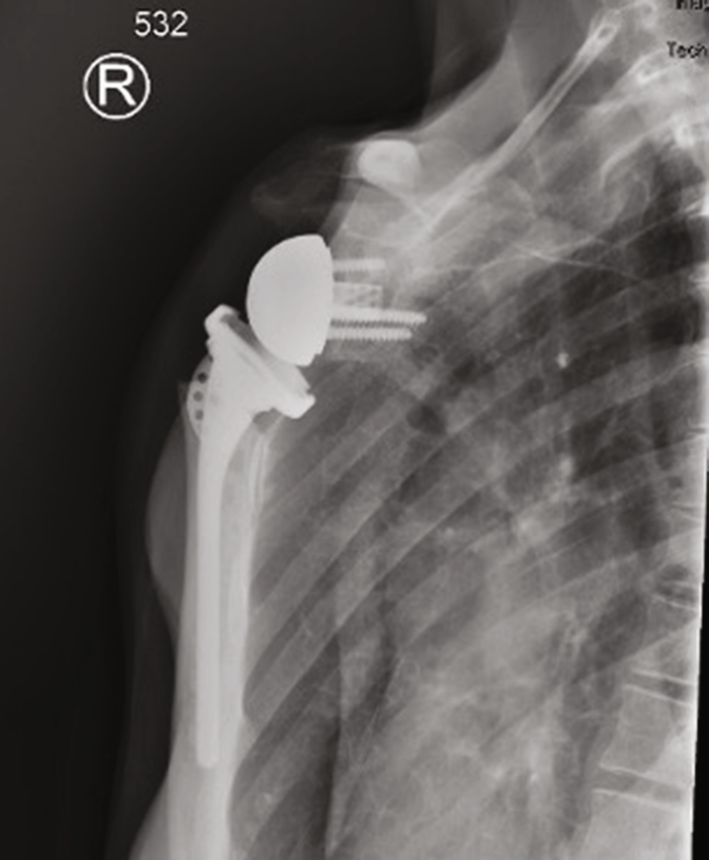
A 1-week postoperative AP radiograph showing appropriate alignment and fixation of the right reverse total shoulder arthroplasty components.

Following the rTSA, the patient's recovery was uneventful. The 1-week postoperative radiographs showed a right rTSA with no evidence of recurrent dislocation (Figure 6). Physical therapy and rehabilitation were initiated to facilitate the restoration of shoulder function and mobility.

**Figure 7:**
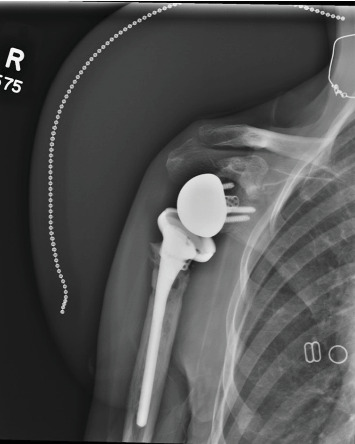
A 6-month postoperative radiograph of the right shoulder showing intact rTSA components with evidence of new osseous irregularity along the humeral stem.

At the 6-month postoperative assessment, radiographs showed a right rTSA with no evidence of fracture and intact surgical hardware (Figure 7). Although the surgical hardware appeared intact, there was evidence of new osseous irregularity along the humeral stem. It was unclear based on imaging if this was a result of infectious etiology or mechanical angiology. Clinically, the patient stated her pain was 6/10, occasionally took Naproxen for pain relief, and was mostly concerned with lack of progress with forward elevation. During the physical exam, the patient had intact sensation to light touch in the C5-T1 dermatomes with no observed deficits. The axilla appeared normal and well perfused. Active range of motion measurements indicated 70° for forward flexion, 70° for abduction, T4 for internal rotation, and 5° for external rotation. Deltoid strength was assessed at 5/5. Patient Reported Outcome Measurement Information System (PROMIS) scores for UE and pain interference (PI) were 36 and 57, respectively. The American Shoulder and Elbow Surgeons Shoulder Score (ASES) was 43.24. The patient affirmed the ability to resume most daily activities. Given the limited range of motion, continued pain, and radiographic evidence of osseous irregularity, the patient's functional outcomes were considered suboptimal for this stage of recovery. In conjunction with the clinical presentation, these findings supported the decision to pursue a right revision rTSA to investigate possible loosening of the humeral prosthesis, add a humeral augment tray and to improve deltoid leverage and range of motion. The patient elected to pursue the procedure due to failed conservative management. During the procedure, there was no evidence of any loosening of the humeral stem and glenoid. Afterwards, there was obvious improvement of the deltoid contour along with the lateral aspect.

**Figure 8:**
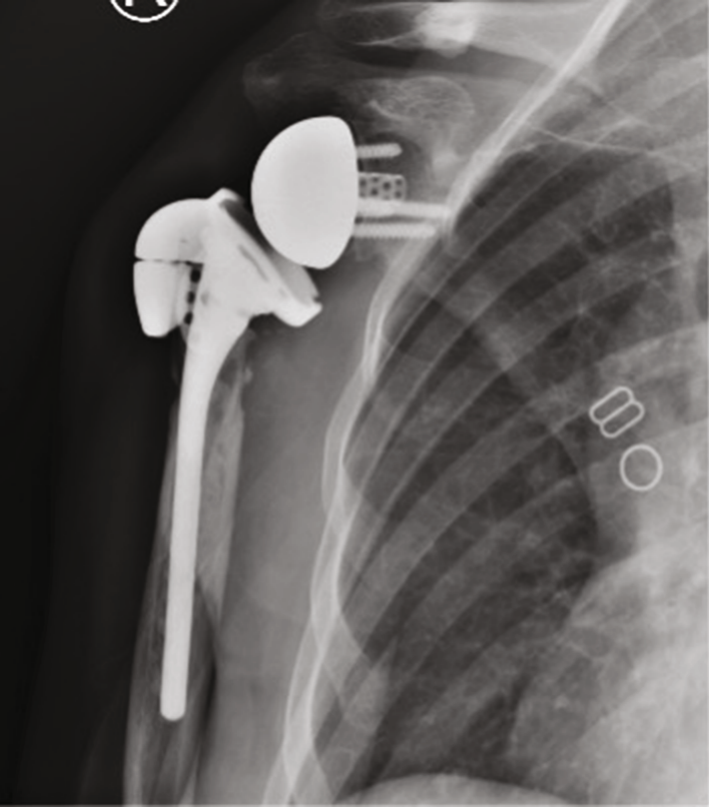
A 1-month follow-up AP radiograph after revision rTSA demonstrating intact hardware and stable prosthetic components.

At the patient's 1-month post-op appointment the patient stated that their pain level was 2/10, denied numbness, tingling, or changes to color on their affected arm. She was taking occasional ibuprofen. The patient said they could put on mascara and do the back of their hair, which they could not perform before her revision. On physical exam of the affected arm, their active flexion was 100°, active abduction was 90°, was able to reach their T6 level with active internal rotation, had active external rotation of 0°, and had passive forward flexion of 140°. Their PROMIS UE and PI scores were 34 and 62, respectively. Their radiographs showed a revised rTSA with intact surgical hardware and without change (Figure 8).

## 3. Discussion

Glenohumeral joint dislocations account for 50% of major joint dislocations, with anterior dislocations being the most prevalent [11]. Although frequent, chronic irreducibility in such cases is relatively uncommon [12], several case reports have highlighted the factors responsible for unsuccessful reduction, such as interposition of the subscapularis muscle, long head of the biceps tendon (LHBT), labrum, greater and lesser tuberosity, and Hill–Sachs impaction on the glenoid [13]. This case is unique in that it involved a chronic irreducible anterior shoulder dislocation complicated by compression of the axillary vein. While vascular complications associated with dislocation typically involve the axillary artery, venous involvement is underreported and often overlooked. To our knowledge, few cases have documented this combination of chronic joint instability and venous compromise confirmed by advanced imaging, necessitating intraoperative vascular surgical planning.

Multiple case reports have described soft tissue interposition as a primary cause of irreducibility. Connolly et al. documented interposition of the subscapularis tendon along with a posteriorly dislocated LHBT [14]. Ayoubi et al. described subscapularis and lesser tuberosity interposition [13], and Pantazis et al. reported LHBT entrapment between the humeral head and a greater tuberosity fragment [15]. These cases reinforce the importance of advanced imaging in chronic dislocations to identify mechanical blocks to reduction.

In all these previous cases, treatment involved rotator cuff repair, LHBT tenodesis, and fracture management through plating or transosseous sutures. However, our approach differed, as we opted for rTSA due to delay of treatment, the patient's three-year rotator cuff tear with fatty atrophy, rendering repair unfeasible, and failure to reduce the joint after multiple attempts. Additionally, our patient presented with an axillary vein compression seen on their CTA. Complications associated with axillary vein compression include deep venous thrombosis, venous insufficiency, UE swelling, and pain [6, 7]. Illig and Doyle comprehensively reviewed Paget–Schroetter syndrome, describing it as effort-induced thrombosis of the subclavian-axillary veins. They emphasize that early diagnosis with duplex or CT/MR venography, followed by prompt thrombolysis and decompression, is essential to prevent complications such as post-thrombotic syndrome [7, 16]. Willis et al. described a similar presentation of upper-extremity deep vein thrombosis following anterior shoulder dislocation and closed reduction, reinforcing that venous thromboses may occur even in cases where reduction appears successful. They recommended prompt Doppler or MR venography when vein injury is suspected to enable timely treatment and prevent serious complications such as pulmonary embolism [17]. In addition to traumatic causes, anatomical variants such as Langer's axillary arch may chronically compress the vein and precipitate intermittent limb swelling. Hafner et al. described such a case, where surgical resection of the muscular band alleviated symptoms [6].

This highlights that nontraumatic compressive processes must also be considered during evaluation for venous compromise. While vascular complications such as axillary artery injury and effort-induced thrombosis (e.g., Paget–Schroetter syndrome) are well documented in the context of acute shoulder dislocation or repetitive motion, there is a notable absence of PubMed-indexed case reports describing chronic axillary vein compression secondary to a persistently dislocated shoulder. The majority of vascular literature focuses on acute arterial events, with venous pathology either secondary to anatomical anomalies like Langer's arch or associated with high-performance activity. To our knowledge, this is the first reported case of chronic axillary vein compression and thrombosis associated with an anterior shoulder dislocation that remained unreduced for several weeks. This highlights the need for vascular imaging in acute traumatic presentations and chronic dislocations where the humeral head may exert prolonged mechanical compression on adjacent vascular structures.

This case underscores the importance of managing chronic, irreducible shoulder dislocations in a tertiary care center rather than an ambulatory surgical center (ASC). Thorough preoperative evaluation, including physical examination and advanced imaging such as MRI and CTA, is essential to assess for osseous injury, rotator cuff incarceration, soft tissue interposition, and neurovascular compromise. In our patient, CTA revealed compression of the axillary vein with associated thrombotic changes, findings that, to our knowledge, have not been previously reported in combination in similar cases. These vascular abnormalities posed a significant risk for intraoperative bleeding, thrombotic propagation, and hemodynamic compromise, warranting intraoperative coordination with vascular surgery. Such high-risk features highlight the necessity of advanced imaging and interdisciplinary planning, typically unavailable in ASCs. Moreover, vascular injury following shoulder dislocation is not limited to venous structures; axillary artery rupture with hemodynamic instability has also been reported in the literature [18]. This reinforces the value of preoperative CTA in chronic dislocation cases, especially when the humeral head lies in close proximity to the neurovascular bundle. Surgical decision-making, facility selection, and multidisciplinary planning are critical to achieving optimal outcomes in these complex scenarios.

While conventional radiographs are the gold standard for confirming shoulder dislocation and assessing osseous structures, one of the most common reasons for irreducible anterior shoulder dislocation is due to soft tissue interposition [19]. A case described by Bridle and Ferris revealed an apparent reduction in an AP radiograph, but the patient persisted with pain [20]. Subsequent open reduction revealed subscapularis interposition within the glenohumeral joint [20]. Therefore, an MRI is an indispensable tool when dealing with chronic shoulder dislocations, as it aids in identifying potential soft tissue interposition, thereby avoiding inadvertent damage to nearby structures when attempting reduction of the joint [20]. Bravman et al. warned that significant vascular injuries can be easily overlooked after low-energy trauma, such as shoulder dislocations, particularly when distal pulses appear intact due to collateral circulation. This underscores the limitations of relying solely on physical examination and plain radiographs, supporting the use of CTA in equivocal or chronic shoulder dislocations [21]. In our case, CTA was pivotal in identifying compromised venous return, prompting preoperative vascular duplex imaging and multidisciplinary planning.

Despite some persistent limitations, the patient's ability to perform previously restricted tasks and reduced PI were meaningful improvements. In a multicenter review by Mahl et al., rTSA performed for chronic locked shoulder dislocations yielded meaningful functional gains; constant scores improved from 13.6 to 47.4, and mean forward flexion increased from 38° to 103°. However, external rotation remained limited (~14.7°), and complications were common, with a 27% revision rate [22]. However, PROMIS scores remained lower than normative values, reflecting the impact of chronic soft tissue degeneration and delayed intervention on functional recovery. Benchmarks from recent literature highlight the responsiveness and validity of PROMIS-UE in shoulder arthroplasty. Gordon et al. reported significant improvements in PROMIS-UE scores at 6 weeks, 12 weeks, and 6 months postoperatively, with excellent correlation to established instruments like ASES, OSS, and SST, and minimal floor/ceiling effects [10]. Our patient's PROMIS-UE score of 20.8 at 3 months was notably lower than expected, underscoring her complex clinical course and delayed recovery. These outcomes align with our case's trajectory and validate our decision to pursue revision surgery. This aligns with the literature on rTSA in complex or revision settings, where full return of motion may not be achievable.

### 3.1. Limitations

This case report is limited by its single-patient design and lack of long-term follow-up beyond the initial postoperative period. Although the diagnosis of axillary vein thrombosis was supported by imaging and intraoperative findings, histologic or flow-based confirmation of chronicity was not obtained. Additionally, while the patient's PROMIS scores provide insight into functional recovery, the absence of a standardized preoperative baseline limits interpretation of postoperative improvement. Finally, generalizability is constrained by the rarity and complexity of this presentation, which may not reflect typical outcomes for chronic shoulder dislocation or venous compression.

## 4. Conclusion

Chronic anterior shoulder dislocations may be complicated by vascular compression, which can be easily overlooked without appropriate imaging. This case underscores the role of CTA in evaluating venous compromise, particularly when the humeral head lies adjacent to the neurovascular bundle. Timely identification of vascular involvement enabled coordination with vascular surgery and informed the decision to proceed with rTSA. Due to the potential for unanticipated vascular compromise, cases of chronic dislocation with suspected neurovascular involvement should be managed in tertiary care centers with vascular surgery availability, not in ambulatory surgical centers. Optimal outcomes require advanced imaging, multidisciplinary evaluation, and surgical planning tailored to the chronicity and complexity of the injury.

## Data Availability

Data sharing does not apply to this article as no datasets were generated or analyzed during the preparation of this case report.
